# Responsible research practices could be more strongly endorsed by Australian university codes of research conduct

**DOI:** 10.1186/s41073-023-00129-1

**Published:** 2023-06-06

**Authors:** Yi Kai Ong, Kay L Double, Lisa Bero, Joanna Diong

**Affiliations:** 1 School of Health Sciences, Faculty of Medicine and Health, The University of Sydney, Sydney, NSW Australia; 2 School of Medical Sciences (Neuroscience), Faculty of Medicine and Health, The University of Sydney, Sydney, NSW Australia; 3 Brain and Mind Centre, The University of Sydney, Sydney, NSW Australia; 4 Center for Bioethics and Humanities, University of Colorado, Aurora, CO USA; 5 School of Medical Sciences (Biomedical Informatics and Digital Health), Faculty of Medicine and Health, The University of Sydney, Sydney, NSW Australia; 6 Charles Perkins Centre, The University of Sydney, New South Wales, Sydney, NSW Australia

**Keywords:** Research integrity, Research quality, Incentives, Regulation, Institution

## Abstract

**Background:**

This study aimed to investigate how strongly Australian university codes of research conduct endorse responsible research practices.

**Methods:**

Codes of research conduct from 25 Australian universities active in health and medical research were obtained from public websites, and audited against 19 questions to assess how strongly they (1) defined research integrity, research quality, and research misconduct, (2) required research to be approved by an appropriate ethics committee, (3) endorsed 9 responsible research practices, and (4) discouraged 5 questionable research practices.

**Results:**

Overall, a median of 10 (IQR 9 to 12) of 19 practices covered in the questions were mentioned, weakly endorsed, or strongly endorsed. Five to 8 of 9 responsible research practices were mentioned, weakly, or strongly endorsed, and 3 questionable research practices were discouraged. Results are stratified by Group of Eight (*n =* 8) and other (*n =* 17) universities. Specifically, (1) 6 (75%) Group of Eight and 11 (65%) other codes of research conduct defined research integrity, 4 (50%) and 8 (47%) defined research quality, and 7 (88%) and 16 (94%) defined research misconduct. (2) All codes required ethics approval for human and animal research. (3) All codes required conflicts of interest to be declared, but there was variability in how strongly other research practices were endorsed. The most commonly endorsed practices were ensuring researcher training in research integrity [8 (100%) and 16 (94%)] and making study data publicly available [6 (75%) and 12 (71%)]. The least commonly endorsed practices were making analysis code publicly available [0 (0%) and 0 (0%)] and registering analysis protocols [0 (0%) and 1 (6%)]. (4) Most codes discouraged fabricating data [5 (63%) and 15 (88%)], selectively deleting or modifying data [5 (63%) and 15 (88%)], and selective reporting of results [3 (38%) and 15 (88%)]. No codes discouraged p-hacking or hypothesising after results are known.

**Conclusions:**

Responsible research practices could be more strongly endorsed by Australian university codes of research conduct. Our findings may not be generalisable to smaller universities, or those not active in health and medical research.

**Supplementary Information:**

The online version contains supplementary material available at 10.1186/s41073-023-00129-1.

## Introduction

High quality research that is rigorous, transparent and reproducible is needed for findings to be valid and trustworthy. Such research is more likely to result in practical and clinical translation of research outcomes and promotes public trust in science. However, the conduct and reporting of research is suboptimal in many disciplines. For example, the prevalence of questionable research practices (e.g. fabricating data, and selectively deleting or modifying data) in many disciplines is high [1, 2]. Approximately 40% of clinical trials on exercise interventions for low back pain do not describe interventions in sufficient detail for independent replication [3]. Moreover, ‘spin’ – the misrepresentation and distortion of research findings – is prevalent in biomedical science [4–6]. Overall, these examples suggest that the validity of scientific claims may, at times, be questionable.

Responsible practices in research conduct and reporting are needed to uphold research integrity and research quality. Responsible research practices often uphold principles of open science and research transparency, and include protocol registration, sharing of de-identified data and computer code, and transparent and rigorous reporting of research. However, there are few strong incentives for responsible research practices in research publishing, academic hiring and promotion, and grant funding [7–9]. Unsurprisingly, the lack of strong incentives may place researchers under pressure to compromise on research quality [10].

University codes of research conduct are documents that govern the behaviour of academic researchers. In Australia, codes of research conduct reflect the Australian Code for the Responsible Conduct of Research (the Australian Code), which outlines principles and responsibilities that both researchers and institutions are expected to follow when conducting research. It is not known to what extent university codes of research conduct endorse responsible research practices. Therefore, this study aimed to investigate how strongly codes of research conduct from research-intensive universities in Australia encourage responsible research practices, and discourage poor research practices.

## Materials and methods

The study protocol and registration are available on the Open Science Framework [11]. Authorship order in this manuscript deviated from authorship order in the registered protocol to reflect author contributions in practice.

### Sampling

We obtained a complete list of Australian universities from the Australian Government website *Study Australia* [12]. We selected 25 universities from this list active in health and medical research, as health and biomedical research receives substantial support from Australian funders [7].

The sample included all Group of Eight universities (*n =* 8), which are Australia’s leading research-intensive universities. Inherently, the Group of Eight is a company incorporated in 1999 that aims to influence the development and delivery of long-term, continual national higher education and research policy, and develop world-class international alliances and research partnerships. Consequently, the Group of Eight is highly influential.

Non-Group of Eight universities (*n =* 17) were sampled from universities reporting high research income awarded by the Australian National Health and Medical Research Council (NHMRC) in 2018, based on data from the Universities Australia Higher Education Research Data Collection [13]. High research income is a key measure to indicate that a university is research-active. Non-Group of Eight universities were purposefully sampled to obtain codes of research conduct from a range of research-active universities with high research income and sizeable public profile. We did not sample universities from which we could not obtain codes of research conduct from public websites (*n =* 2). Our final sample consisted of 8 Group of Eight and 17 non-Group of Eight universities.

Codes of research conduct and related documents were obtained from the public websites of the 25 included universities. The list of included universities is available in the Additional file 1.

### Question development and pilot testing

The Australian Code outlines principles and responsibilities that researchers and institutions are expected to follow when conducting research. It includes more detailed guides on specific aspects of responsible research conduct. We interpreted how the 2018 Australian Code and its supporting guides could be translated into practice, and developed 19 questions and scoring criteria to assess if university codes of research conduct defined important aspects of research integrity, and how the codes advised or required researchers to implement responsible research practices. The questions were developed using questions from our previous audit of incentives for responsible research practices in grant funding schemes [7], and recommendations by large consensus papers [9, 14]. Questions assessed if codes of research conduct defined research integrity, research quality, and research misconduct (Q1-3, outcomes scored as *No*: not mentioned, *Default*: the code defaults to the Australian Code, *Specific*: the code states its own definition), required research to be approved by an appropriate ethics committee (Q4-5, outcomes scored as *No*, *Yes*), endorsed (i.e. advised or required) responsible research practices (Q6-14), and discouraged questionable research practices (Q15-19, outcomes in this and the preceding section scored as *No*: not mentioned, *Default*: the code defaults to the Australian Code, *Advised*: implies strongly recommended but not mandated; a weak endorsement, *Required*: implies mandatory, likely with penalties if violated; a strong endorsement).

Questions were scored as ‘Advised’ if codes of research conduct used words such as ‘advise’, ‘suggest’, ‘should’, ‘encourage’, and ‘recommend’. Questions were scored as ‘Required’ if codes of research conduct used words such as ‘require’, ‘mandate’, and ‘must’. The word ‘should’ was only used to score a question as ‘required’ if the code of research conduct used this word and stated penalties that researchers would face if they did not comply. The wording of the questions and scoring criteria are shown in Table 1.

**Table 1:** Questions to assess how strongly university codes of research conduct (1) defined research integrity, quality and misconduct, (2) required ethics approval, (3) endorsed 9 responsible research practices and (4) discouraged 5 research misbehaviours and questionable research practices. Variable names used in Fig. 1 are in *italics*

**Definitions.** **Does the code define:**	
'research integrity'?	*research integrity*
'research quality'?	*research quality*
'research misconduct'?	*research misconduct*
**Ethics approval.** **Does the code state that:**	
all research on humans must be approved by an appropriate ethics committee?	NA
all research on animals must be approved by an appropriate ethics committee?	NA
**Responsible research practices.** **Does the code state that:**	
study protocols of clinical trials should be publicly registered?	*register trial protocol*
study protocols of other study designs should be publicly registered?	*register other protocol*
analysis protocols should be publicly registered?	*register analysis protocol*
study data should be made publicly available?	*open data*
analysis code should be made publicly available?	*open code*
findings should be published on open access platforms?	*open publishing*
reporting guidelines (e.g. from journals, professional associations, or the EQUATOR network) should be used in reporting research?	*reporting guidelines*
conflicts of interest should be declared?	*conflicts of interest*
researchers should receive training in research integrity and other areas (e.g. research supervision, data management, peer review, publishing 'negative' findings)?	*researcher training*
**Research misbehaviours and questionable research practices.** **Does the code state that:**	
fabricating data should be discouraged?	*fabricate data*
selectively deleting or modifying data after performing initial data analysis should be discouraged?	*select data*
selectively reporting results (e.g. not publishing a valid 'negative' finding) should be discouraged?	*select results*
performing analyses until statistically significant results are obtained (i.e. p should be discouraged?	*p-hacking*
hypothesizing after results are known should be discouraged?	*harking*

Five university codes of research conduct were randomly sampled and used in a pilot study to assess the clarity of scoring instructions and agreement between investigators. Feedback from the pilot study was used to refine the wording of the questions and scores.

For the full audit, the text of the codes of research conduct and related documents was independently scored by 3 investigators (YKO, JD, KD). Scores that differed between investigators were discussed to reach agreement by consensus and refine the questions and scoring criteria. The refined questions and criteria were applied to the full sample (which included codes of research conduct used in the pilot study).

### Document retrieval and data collection

University codes of research conduct and related documents referenced in hyperlinks within each code were retrieved by a single investigator (YKO) within 1 week (14–18 Jan 2021). The following procedure was applied to retrieve documents:

- The main website or document of the university code of research conduct was first retrieved using search terms such as ‘research conduct’, ‘code of research conduct’, ‘responsible research’ and ‘research code’.
- All documents referenced in hyperlinks from the main website or document of the code of research conduct were retrieved; we refer to this as Level 1 information.
- Level 1 documents were screened. Only relevant documents referenced in hyperlinks in Level 1 documents were retrieved; we refer to this as Level 2 information.

When related documents could not be retrieved from a university’s public website, we contacted the university to request them. We were not able to retrieve the Research Ethics Manual referenced in the code of research conduct for one university. We contacted the university’s ethics board and they supplied us with an internal copy of the document.

Codes of research conduct and related documents were audited by a single investigator (YKO) and checked by another investigator (JD), using the questions and scoring criteria. Potential errors in scoring or lack of agreement were discussed with the investigator team and resolved by consensus. Decision rules to score questions are available in the Additional file 1.

A separate audit of the Australian Code and its supporting guides against our questions was also performed.

### Analysis

Descriptive data are reported. All data processing and analysis were performed using Python (v3.9). Data and computer analysis code are available from the project repository [15].

## Results

Codes of research conduct from 25 universities were audited (full list in Additional file 1). Based on HERDC data, the median (IQR) NHMRC research income obtained by these universities in 2018 was AUD $8.3 ($4.4 to $26.5) million.

**Fig. 1:**
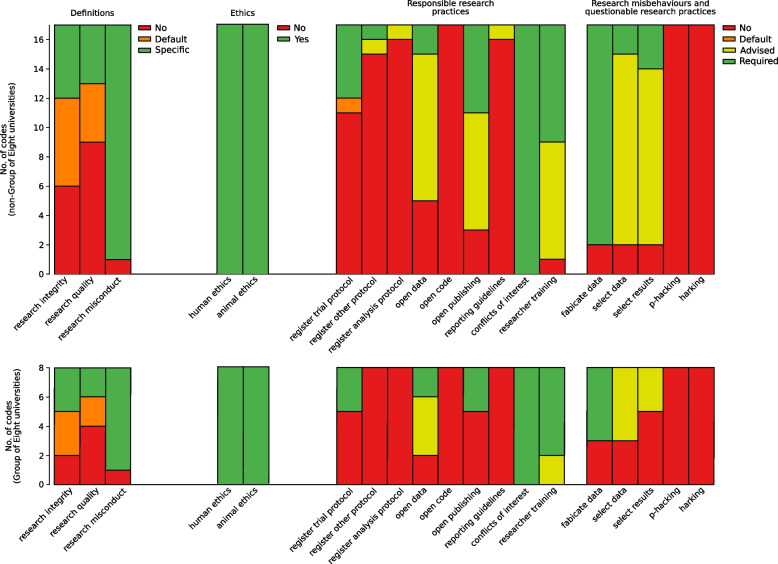
Number of university codes of research conduct that (1) defined research integrity, quality and misconduct; outcomes in this section were scored as *No*: not mentioned, *Default*: the code defaults to the Australian Code, *Specific*: the code states its own definition, (2) required ethics approval; outcomes in this section were scored as *No* and *Yes*, (3) endorsed responsible research practices and (4) discouraged research misbehaviours and questionable research practices; outcomes in sections (3) and (4) were scored as *No*: not mentioned, *Default*: the code defaults to the Australian Code, *Advised*: implies strongly recommended but not mandated (a weak endorsement), *Required*: implies mandatory, likely with penalties if violated (a strong endorsement). Refer to Table 1 for wording of questions and variable names in this figure

The number of codes of research conduct that satisfied the criteria for each of the questions is shown in Fig. 1. Results are stratified by Group of Eight (*n =* 8) and non-Group of Eight (*n =* 17) universities. Counts and percentages of scores for each university code of research conduct for each question are available in the Additional file 1.

Overall, a median of 10 (IQR 9 to 12) of 19 practices covered in the questions were mentioned (i.e. the code of research conduct defaulted to the Australian Code), weakly endorsed, or strongly endorsed. Specifically, 7 (88%) Group of Eight and 16 (94%) non-Group of Eight codes of research conduct defined research misconduct, however only 6 (75%) and 11 (65%) defined research integrity, and 4 (50%) and 8 (47%) defined research quality. Twenty-four to 38% of codes of research conduct across Group of Eight and non-Group of Eight universities defined research integrity and research quality by defaulting to the Australian Code.

All codes of research conduct required ethics approval for human and animal research.

Overall, 5 to 8 of 9 responsible research practices were mentioned, weakly endorsed, or strongly endorsed, and 3 questionable research practices were discouraged. Specifically, all codes of research conduct required conflicts of interest to be declared, but there was substantial variation for other responsible research practices. The most commonly endorsed responsible research practices were ensuring researcher training in research integrity and other areas [8 (100%) and 16 (94%)], and making study data publicly available [6 (75%) and 12 (71%)]. The least commonly endorsed responsible research practices were making analysis code publicly available [0 (0%) and 0 (0%)], registering analysis protocols [0 (0%) and 1 (6%)], using reporting guidelines in reporting research [0 (0%) and 1 (6%)], and registering study protocols of study designs other than clinical trials [0 (0%) and 2 (12%)]. Proportionally, compared to Group of Eight universities, more non-Group of Eight universities endorsed that study protocols of designs other than clinical trials be registered, and findings be published on open access platforms.

Most codes of research conduct discouraged fabricating data [5 (63%) and 15 (88%)], selectively deleting or modifying data [5 (63%) and 15 (88%)], and selectively reporting results [3 (38%) and 15 (88%)]. Proportionally, compared to Group of Eight universities, more non-Group of Eight universities mandated against selectively deleting or modifying data and selective reporting of results. No codes of research conduct discouraged p-hacking or hypothesising after results are known (harking).

Results of the audit of the Australian Code and its supporting guides against our questions are available in the Additional file 1. Overall, in the Australian Code and its supporting guides, ‘research integrity’ and ‘research misconduct’ were defined, but ‘research quality’ was not. The Australian Code required ethics approval for human and animal research. Eight of 9 responsible research practices were mentioned, weakly endorsed, or strongly endorsed, and 1 of 5 questionable research practices was discouraged.

Lists of all documents accessed and included for each university (including number, version, year of publication) are also available in the Additional file 1.

## Discussion

Australian university codes of research conduct strongly endorse some aspects of research integrity (e.g. requiring ethics approval, declaring conflicts of interest), as outlined in principle by the Australian Code for the Responsible Conduct of Research. However, other responsible research practices are weakly endorsed, or are not mentioned (e.g. registering protocols, open code, discouraging p-hacking). In general, all codes of research conduct could endorse responsible research practices more strongly, and discourage questionable research practices more explicitly.

We sampled to include codes of research conduct from Australian universities active in health and medical research, based on research income awarded by the NHMRC. Thus, our findings would best reflect how strongly codes of research conduct from these universities encouraged responsible research practices, and discouraged questionable research practices. This is important because substantial research efforts in health and biomedical research are supported by the Australian government. In 2019–2020, approximately AUD $202.5 billion was spent on health goods and services in Australia. This comprised 10.2% of overall economic activity [16], with large funds channeled through the NHMRC (e.g. AUD $870 million in 2021–2022) [17] and other funders. Researchers supported by these funders may be more likely to apply responsible research practices (or refrain from poor research practices) if their university’s code of research conduct explicitly endorse them.

Why do codes of research conduct not endorse responsible research practices more strongly, or not at all? First, substantial efforts to date have been directed towards preventing or minimising research misconduct and fraud, as these have broad, detrimental ramifications on public trust in science [18, 19]. Other poor research practices such as p-hacking or hypothesising after results are known may be regarded as less severe, and not prioritised. Compared to misconduct and fraud, upholding research integrity and quality through positive measures can seem less urgent, and more laborious to implement. Second, responsible research practices could be viewed by universities to fall under the remit of individual researchers, not institutions. Thus, universities may dismiss the need to endorse these practices more strongly. Third, universities might fail to adequately understand the value and need for responsible research practices, especially in context of the pressure to publish. Overall, these barriers may impede the broader implementation of responsible research practices in research conduct and reporting.

Codes of research conduct could endorse responsible research practices more strongly in several ways. Codes of research conduct could be regularly updated to mandate responsible research practices or hold researchers more accountable, especially when current wording simply defaults to the Australian Code, or only serves as a weak endorsement. Codes of research conduct could also be written to clearly attribute responsibility either to individuals or to institutions [20], or explicitly define good research ‘culture’ and how it can be developed. This could decrease reliance on research culture to drive the uptake of responsible research practices. More broadly, future research could investigate other strategies or themes to inform codes of research conduct and incentives for responsible research practices. Examples of these include the responsible evaluation of research and researchers, how open science and transparency impact responsible research practices, and how responsible mentoring, supervision and role modelling could be conducted [21]. Importantly, these efforts would allow codes of research conduct to implement policies that are evidence based [22].

Our findings should be interpreted in context of study limitations. First, we purposefully sampled codes of research conduct from universities active in health and medical research. This may mean our findings are not generalisable to smaller universities, universities that focus on other research areas, or universities that are not research-intensive. Second, document retrieval was difficult at times as some documents were not publicly accessible, or had multiple embedded hyperlinks. We contacted university research offices to request copies of documents that were not accessible. The lack of public access to these documents seems problematic: it may suggest universities do not highly prioritise transparency in the governance of research processes. Third, instructions on specific practices (e.g. use reporting guidelines, discourage p-hacking and harking) may be present in internal university documents to relevant faculties, even though they were not stated in externally-facing documents to the University that could be accessed publicly. By auditing only the externally-facing documents, we would not have been able to capture internally-available information that is relevant to research conduct. Fourth, statements in some codes were ambiguous and difficult to interpret. In such instances, we endeavoured to make reasonable decision rules for interpretation, and we recorded the rationale for these decisions to ensure consistent assessment of subsequent codes of research conduct.

## Conclusions

In summary, Australian university codes of research conduct could endorse responsible research practices more strongly, and discourage questionable research practices more explicitly. These measures could be implemented by clearly attributing responsibility for research rigour to individual researchers or to universities through the codes of research conduct. If the global scientific community collectively identifies and implements good research practices, in time, this may improve research integrity and research quality, and improve trustworthiness in science.

## Supplementary Information

**Additional file 1.**
