# Supplementary material for: Responsible research practices could be more strongly endorsed by Australian university codes of research conduct
Source: Res Integr Peer Rev. 2023 Jun 6;8:5. doi: 10.1186/s41073-023-00129-1 (PMC10242962; doi:10.1186/s41073-023-00129-1)
Supplement: Supplementary file 1 — Additional file 1. [file 41073_2023_129_MOESM1_ESM.pdf]

Appendix

Table of Contents

List of included universities 2

Decision rules 3

Counts and percentages of scores for each university code of research conduct 5

Audit of the Australian Code for the Responsible Conduct of Research 12

Documents assessed and included for each university 17

List of included universities

Australian Catholic University
Australian National University*
Bond University
Curtin University
Deakin University
Flinders University
Griffith University
James Cook University
Macquarie University
Monash University*
Murdoch University
Queensland University of Technology
Royal Melbourne Institute of Technology
The University of Western Australia*
The University of Adelaide*
The University of Melbourne*
The University of Queensland*
The University of Sydney*
University of Canberra
University of New South Wales*
University of Newcastle
University of Tasmania
University of Technology Sydney
University of Wollongong
Western Sydney University

*Group of Eight University

Decision rules

Scoring often involved making qualitative judgements of the wording in the codes of research conduct. In the following instances, we endeavoured to grant benefit of doubt where wording was ambiguous. For example,

Q9. Does the code state that study data should be made publicly available?

Several codes of research conduct only instructed investigators to make study data available to other researchers, not to the public. Examples:

- Deakin University, Research Data and Primary Materials Policy, clause 23: 'Researchers should make research data available for the use by other researchers.'
- University of New South Wales, Research Data Governance and Materials Handling Policy, section 5: 'Researchers must make available any research data and materials related to publications for discussion with other researchers.'

For these codes, we established the decision rule to score this question as 'No'.

Q14. Does the code state that researchers should receive training in research integrity and other areas (e.g. research supervision, data management, peer review, publishing 'negative' findings)?

Some codes of research conduct stated that research integrity training applies to one group of researchers (e.g. research students), without referencing other groups of researchers. Example:

- University of Adelaide, 2021 Research Student Handbook, slide 27: '… satisfactory completion requires that you have: completed training in research integrity and have an understanding of University and Australian policies on the responsible conduct of research, including avoiding plagarism.'

For this code, we interpreted this requirement as extending to all researchers, and scored this question as 'Required'.

In addition, some codes of research conduct state that research training is mandatory for new staff or students, without stating whether it is mandatory for existing staff. Examples:

- University of Canberra, Responsible Conduct of Research Policy, section 3.3: 'The completion of Research Integrity training will be a mandatory requirement for all new academic staff as well as HDR students; Research Integrity training will also be recommended for existing academic staff;'
- Curtin University, Research Management Policy, section 2.4.2: 'Staff and Higher Degree by Research students undertaking research will complete the Research Integrity provided by the University within six months of appointment for staff or prior to completion of milestone 1 for Higher Degree by Research students.'

For these codes, we felt compelled to interpret their recommendation less strongly as it was not consistent across all groups of researchers. Thus, we scored this question as ‘Advised’.

Does the code state that the following research misbehaviours and questionable research practices should be discouraged? Q16. Selectively deleting or modifying data after performing initial data analysis; Q17. Selectively reporting results (e.g. not publishing a valid ‘negative’ finding).

Some codes of research conduct did not explicitly discourage selectively deleting data or the selective reporting of results. Others discouraged ‘falsification or misrepresentation of research data’. Example:

- Murdoch University, Research Integrity Policy, Attachment 1: ‘Examples of research integrity breaches include, but are not limited to, the following: ii. Fabrication, falsification, misrepresentation: Fabrication of research data or source material; Falsification of the research data or source material; Misrepresentation of research data or source material; Falsification and/or misrepresentation to obtain funding.’

For this code, we interpreted these statements to broadly cover other misbehaviours such as selectively deleting data and selectively reporting results. Thus, we scored this question as ‘Advised’.

Counts and percentages of scores for each university code of research conduct

DEFINITIONS

Q1. Does the code define ‘research integrity’?

Non-Group of Eight
default 6
no 6
specific 5

default 35.3
no 35.3
specific 29.4

Group of Eight
default 3
no 2
specific 3

default 37.5
no 25.0
specific 37.5

Q2. Does the code define ‘research quality’?

Non-Group of Eight
default 4
no 9
specific 4

default 23.5
no 52.9
specific 23.5

Group of Eight
default 2
no 4
specific 2

default 25.0
no 50.0
specific 25.0

Q3. Does the code define ‘research misconduct’?

Non-Group of Eight
no 1
specific 16

no 5.9
specific 94.1

Group of Eight
no 1
specific 7

no 12.5
specific 87.5

ETHICS

Q4. Does the code state that all research on humans must be approved by an appropriate Ethics Committee?

Non-Group of Eight
yes 17

yes 100.0

Group of Eight
yes 8

yes 100.0

Q5. Does the code state that all research on animals must be approved by an appropriate Ethics Committee?

Non-Group of Eight
yes 17

yes 100.0

Group of Eight
yes 8

yes 100.0

RESPONSIBLE PRACTICES AND MISBEHAVIOURS

Q6. Does the code state that study protocols of clinical trials should be publicly registered?

Non-Group of Eight
default 1

no 11
required 5

default 5.9
no 64.7
required 29.4

Group of Eight
no 5
required 3

no 62.5
required 37.5

Q7. Does the code state that study protocols of other study designs should be publicly registered?

Non-Group of Eight
advised 1
no 15
required 1

advised 5.9
no 88.2
required 5.9

Group of Eight
no 8

no 100.0

Q8. Does the code state that analysis protocols should be publicly registered?

Non-Group of Eight
advised 1
no 16

advised 5.9
no 94.1

Group of Eight
no 8

no 100.0

Q9. Does the code state that study data should be made publicly available?

Non-Group of Eight
advised 10
no 5
required 2

advised 58.8
no 29.4
required 11.8

Group of Eight
advised 4
no 2
required 2

advised 50.0
no 25.0
required 25.0

Q10. Does the code state that analysis code should be made publicly available?

Non-Group of Eight
no 17

no 100.0

Group of Eight
no 8

no 100.0

Q11. Does the code state that findings should be published on open access platforms?

Non-Group of Eight
advised 8
no 3
required 6

advised 47.1
no 17.6
required 35.3

Group of Eight
no 5
required 3

no 62.5
required 37.5

Q12. Does the code state that reporting guidelines (e.g. from journals, professional associations, or the EQUATOR network) should be used in reporting research?

Non-Group of Eight
advised 1
no 16

advised 5.9
no 94.1

Group of Eight
no 8

no 100.0

Q13. Does the code state that conflicts of interest should be declared?

Non-Group of Eight
required 17

required 100.0

Group of Eight
required 8

required 100.0

Q14. Does the code state that researchers should receive training in research integrity and other areas (e.g. research supervision, data management, peer review, publishing 'negative' findings)?

Non-Group of Eight
advised 8
no 1
required 8

advised 47.1
no 5.9
required 47.1

Group of Eight
advised 2
required 6

advised 25.0
required 75.0

Q15. Does the code state that fabricating data should be discouraged

Non-Group of Eight
no 2
required 15

no 11.8
required 88.2

Group of Eight
no 3
required 5

no 37.5
required 62.5

Q16. Does the code state that selectively deleting or modifying data after performing initial data analysis should be discouraged

Non-Group of Eight
advised 13
no 2
required 2

advised 76.5
no 11.8
required 11.8

Group of Eight
advised 5
no 3

advised 62.5
no 37.5

Q17. Does the code state that selectively reporting results (e.g. not publishing a valid ‘negative’ finding) should be discouraged

Non-Group of Eight
advised 12
no 2
required 3

advised 70.6
no 11.8
required 17.6

Group of Eight
advised 3
no 5

advised 37.5
no 62.5

Q18. Does the code state that performing analyses until statistically significant results are obtained (i.e. p-hacking) should be discouraged

Non-Group of Eight
no 17

no 100.0

Group of Eight
no 8

no 100.0

Q19. Does the code state that hypothesizing after results are known should be discouraged

Non-Group of Eight
no 17

no 100.0

Group of Eight
no 8

no 100.0

Audit of the Australian Code for the Responsible Conduct of Research

The template for the audit of university codes of research conduct is used. Original scoring criteria are retained.

For outcomes in the following section,
"Default": means the University code defaults to the Australian Code of Research Conduct
"Specific": means the University code states its own definition

***Definitions*. Does the University code of research conduct (referred to as the "code" hereafter) define:**

"research integrity"?
Yes, specific – record statement / Yes, default / No
Principles P1-P8
– Main document

"research quality"?
Yes, specific – record statement / Yes, default / **No**

"research misconduct"?
Yes, specific – record statement / Yes, default / No
"Research misconduct. A serious breach of the Code which is also intentional or reckless or negligent."
– Main document

Comments on definitions

***Ethics*. Does the code state that:**

all research on humans must be approved by an appropriate Ethics Committee?
Yes – record statement / No
"2.1 i. Not meeting required research standards
• Conducting research without ethics approval as required by the National Statement on Ethical Conduct in Human Research and the Australian Code for the Care and Use of Animals
for Scientific Purposes
• Failing to conduct research as approved by an appropriate ethics review body"
– Guide to Managing and Investigating Potential Breaches of the Australian Code for the Responsible Conduct of Research

all research on animals must be approved by an appropriate Ethics Committee?
Yes – record statement / No
"2.1 i. Not meeting required research standards
• Conducting research without ethics approval as required by the National Statement on Ethical Conduct in Human Research and the Australian Code for the Care and Use of Animals
for Scientific Purposes
• Failing to conduct research as approved by an appropriate ethics review body"
– Guide to Managing and Investigating Potential Breaches of the Australian Code for the Responsible Conduct of Research

Comments on ethics

For outcomes in the following section,
"Default": means the University code defaults to the Australian Code of Research Conduct
"Advised": implies strongly recommended but not mandated; a weak incentive
"Required": implies mandatory, likely with penalties if violated; a strong incentive

***Requirements for good scientific practices.* Does the code state that:**

study protocols of clinical trials should be publicly registered?
Yes, required – record statement / Yes, advised – record statement / Yes, default / No
"4.6 For any research project that prospectively assigns participants to one or more health-related
interventions to evaluate the effects on health outcomes, researchers must register the project
as a clinical trial on a publicly accessible register complying with international standards before the recruitment of the first participant."
– Publication and dissemination of research

study protocols of other study designs should be publicly registered?
Yes, required – record statement / **Yes, advised** – record statement / Yes, default / No
"4.6 Other human and animal research also can be registered on international databases."
– Publication and dissemination of research

analysis protocols should be publicly registered?
Yes, required – record statement / **Yes, advised** – record statement / Yes, default / No
"4.6 Researchers should, where appropriate, consider registering their research plans or protocols
prior to the commencement of research."

– Publication and dissemination of research

study data should be made publicly available?
Yes, required – record statement / **Yes, advised** – record statement / Yes, default / No
"R22 Retain clear, accurate, secure and complete records of all research including research data and
primary materials. Where possible and appropriate, allow access and reference to these by interested parties."
– Main document
"4.6 At the conclusion of a project, and where possible and appropriate, researchers should publish or allow interested parties to access and refer to research data, survey instruments, coding manuals and the tools and resources that supported analysis of research data."
– Publication and dissemination of research

analysis code should be made publicly available?
Yes, required – record statement / **Yes, advised** – record statement / Yes, default / No
"4.6 At the conclusion of a project, and where possible and appropriate, researchers should publish or allow interested parties to access and refer to research data, survey instruments, coding manuals and the tools and resources that supported analysis of research data."
– Publication and dissemination of research

findings should be published on open access platforms?
Yes, required – record statement / **Yes, advised** – record statement / Yes, default / No
"4.6 At the conclusion of a project, and where possible and appropriate, researchers should publish or allow interested parties to access and refer to research data, survey instruments, coding manuals and the tools and resources that supported analysis of research data."
– Publication and dissemination of research

> if Yes,
> Is there University funding to support open access publication?
> Yes – record statement/ No / **NA**

reporting guidelines (e.g. from journals, professional associations, or the EQUATOR network) should be used in reporting research?
Yes, required – record statement / Yes, advised – record statement / Yes, default / **No**
"Footnote 3. See also reporting guidelines at http://equator-network.org."
(Reporting guidelines were mentioned, but no specific instruction to endorse)
– Publication and dissemination of research

conflicts of interest should be declared?
Yes, required – record statement / Yes, advised – record statement / Yes, default / No
"R24 Disclose and manage actual, potential or perceived conflicts of interest."
– Main document

researchers should receive training in research integrity and other areas (e.g. research supervision, data management, peer review, publishing “negative” findings)?
Yes, required – record statement / Yes, advised – record statement / Yes, default / No
“R4 Provide ongoing training and education that promotes and supports responsible research
conduct for all researchers and those in other relevant roles.”
“R16 Undertake and promote education and training in responsible research conduct.”
– Main document

the following research misbehaviours and questionable research practices should be discouraged?

- fabricating data
  Yes, required – record statement / Yes, advised – record statement / Yes, default / No
  “2.1 ii Fabrication, falsification, misrepresentation
  • Fabrication of research data or source material”
  – Guide to Managing and Investigating Potential Breaches of the Australian Code for the Responsible Conduct of Research

- selectively deleting or modifying data after performing initial data analysis
  Yes, required – record statement / Yes, advised – record statement / Yes, default / **No**
  “2.1 ii Fabrication, falsification, misrepresentation
  • Misrepresentation of research data or source material”
  (No specific instruction to discourage this item)
  – Guide to Managing and Investigating Potential Breaches of the Australian Code for the Responsible Conduct of Research

- selectively reporting results (e.g. not publishing a valid “negative” finding)
  Yes, required – record statement / Yes, advised – record statement / Yes, default / **No**
  “2.1 ii Fabrication, falsification, misrepresentation
  • Misrepresentation of research data or source material”
  (No specific instruction to discourage this item)
  – Guide to Managing and Investigating Potential Breaches of the Australian Code for the Responsible Conduct of Research

- performing analyses until statistically significant results are obtained (i.e. p-hacking)
  Yes, required – record statement / Yes, advised – record statement / Yes, default / **No**
  “2.1 ii Fabrication, falsification, misrepresentation

• Misrepresentation of research data or source material”
(No specific instruction to discourage this item)
– Guide to Managing and Investigating Potential Breaches of the Australian Code for the Responsible Conduct of Research

- hypothesizing after results are known
  Yes, required – record statement / Yes, advised – record statement / Yes, default / **No**
  “2.1 ii Fabrication, falsification, misrepresentation
  • Misrepresentation of research data or source material”
  (No specific instruction to discourage this item)
  – Guide to Managing and Investigating Potential Breaches of the Australian Code for the Responsible Conduct of Research

Comments on requirements for good scientific practices

Documents assessed and included for each university

No.	Document	Version	Year
**U1**	**Australian Catholic University**	Unavailable	Unavailable
1.	Academic Integrity and Misconduct Policy	Unavailable	Unavailable
2.	ACU higher_degree_research_regulations	Unavailable	Unavailable
3.	ACU_Research_Code_of_Conduct	Unavailable	2019
4.	ACU Retention and Disposal Schedule 2013	Unavailable	2013
5.	Australian Catholic University Reseach Funding (ACURF) Guideline	2	2016
6.	Complaints and Investigations Procedure 20201015	1	2020
7.	Higher_Degree_Research_Supervision_Policy	Unavailable	2020
8.	HREC Peer Review Policy	Unavailable	2011
9.	IP_Policy_Final	Unavailable	2014
10.	Policy11CodeofConductandDisciplinePolicyandProcedurev15	1.5	2018
11.	Privacy_Policy_Final_approved_18	Unavailable	2018
12.	Reseach Publication Policy	1	2014
13.	Research_Authorship_Policy_20140507	1	2014
14.	Research_Data_Management_Policy	Unavailable	2017
**U2**	**Australian National University**		
1.	Research misconduct and serious research misconduct	13	2015
2.	Animal Ethics Overview	Unavailable	Unavailable
3.	Conduct of Research	15	2020
4.	Human Research Ethics Committee	Unavailable	Unavailable
5.	Intellectual property Policy	10	2017
6.	Open Access Policy	6	2020
7.	Privacy Policy	10	2015
8.	Public interest disclosure	6	2014
9.	Responding to allegations of scientific misconduct involving US PHS research grants	4	2004
10.	Responsible Conduct of Research	8	2019
**U3**	**Bond University**		
1.	Academic Integrity Policy	1	2020

No.	Document	Version	Year
2.	Authorship Policy	1	2020
3.	Bond Instituitional Biosafety Policy	1	2019
4.	Bond University Animal Research Ethics Committee (BUAREC) Policy	1	2020
5.	Bond University Human Research Ethics Policy	3	2020
6.	Copyright compliance policy	6	2019
7.	Defence EUnavailableport Control Policy	2	2019
8.	Higher Degree Research Student Supervision Policy	3	2019
9.	Bond University Human Reseach Ethics Policy	3	2020
10.	Intellectual Property Policy	1	2019
11.	Open Access Policy	2	2020
12.	Research Code of Conduct Policy	4	2020
13.	Research data management and sharing policy	2	2020
14.	Research Misconduct Policy	2	2019
**U4**	**Curtin University**		
1.	Accessible Information Procedures	Unavailable	2020
2.	Authorship, Peer Review and Publication of Research Outputs Policy	1	2020
3.	Authorship, Peer Review and Publication of Research Outputs Procedures	1	2020
4.	Curtin University Code of conduct	Unavailable	2019
5.	Conflict of Interest Procedures	1	2016
6.	Intellectual Property Policy	1.2	2017
7.	Intellectual Property Procedures	Unavailable	2019
8.	Records and Information Management Procedures	1.6b	2016
9.	Research Data and Primary Materials Policy	1	2020
10.	Research Management Policy	1	2020
11.	Responsible Conduct of Research (Staff) Procedures	1	2020
12.	Responsible Conduct of Research Policy	1	2020
**U5**	**Deakin University**		
1.	Conflict of Interest Procedure	Unavailable	2020
2.	EUnavailableternal Relationships policy	Unavailable	2016

No.	Document	Version	Year
3.	EUnavailableternally Funded Research Contracts Procedures	Unavailable	2016
4.	Higher Degrees by Research (HDR) policy	Unavailable	2019
5.	Higher Degrees by Research (HDR) Supervision procedure	Unavailable	2019
6.	Publication and Dissemination of Research procedure	Unavailable	2016
7.	Regulation 5.2.2 Higher Education Award Courses General	Unavailable	2020
8.	Regulation 9.1(1) Intellectual Propertty	Unavailable	2012
9.	Research Authorship procedure	Unavailable	2020
10.	Research Conduct Policy	Unavailable	2020
11.	Research Data and Primary Materials Management procedure	Unavailable	2016
12.	Research Integrity Breaches procedure	Unavailable	2020
13.	Research Peer Review procedure	Unavailable	2020
14.	Statue 2.2 The Academic Board	Unavailable	2011
15.	Statue 5.2 Academic Awards	Unavailable	2008
16.	Statue 9.1 Intellectual Property	Unavailable	2012
**U6**	**Flinders University**		
1.	Animal Ethics	Unavailable	Unavailable
2.	Breach of Research Integrity Procedures	Unavailable	2019
3.	Conflict of Interest Policy	Unavailable	2017
4.	Charter for HDR Student and Supervisor Responsibilities	Unavailable	2018
5.	Human ethics	Unavailable	Unavailable
6.	Management of Research Data and Primary Materials Policy	Unavailable	2016
7.	Research Integrity Policy	Unavailable	2019
8.	Research Publication, Authorship and Peer Review Policy	Unavailable	2016
**U7**	**Griffith University**		
1.	Academic Misconduct Policy – Higher Degree Research Candidates	Unavailable	2019
2.	Bachelor Honours Degree (AQF Level 8) Policy	Unavailable	2017
3.	Code of Practice for the Supervision of Higher Degree Research Candidates	Unavailable	2019
4.	Conflict of Interest Policy	Unavailable	2020
5.	Consultancy and Commercial Research Policy	Unavailable	2020

No.	Document	Version	Year
6.	Professional and Support Staff Enterprise Agreement 2017-2021	Unavailable	2017
7.	Guidelines for Animal Care and Use in Teaching and Research	Unavailable	2019
8.	Guidelines for Undertaking a Dissertation in Bachelor (Honours), Graduate Diploma and Masters Degree Programs	Unavailable	2018
9.	Higher Degree Research Supervisor Accreditation Policy	Unavailable	2018
10.	Higher Degree Research Policy	Unavailable	2020
11.	Intellectual Property Policy	Unavailable	2017
12.	Private Practice Policy	Unavailable	2017
13.	Records Management Policy	Unavailable	2018
14.	Research Integrity Breach Investigation Procedure	Unavailable	2019
15.	Role Statement HDR Convenor	Unavailable	2013
16.	Role Statement Honours Program Director	Unavailable	2014
17.	Student Misconduct Policy	Unavailable	2019
18.	The Responsible Conduct of Research	Unavailable	2019
19.	Griffith University Research Ethics Manual	3.08	2020
**U8**	**James Cook University**		
1.	Authorship Procedure	19-1	2019
2.	Code for the Responsible Conduct of Research	19-1	2019
3.	Collaborative Research Procedure	20-1	2020
4	Disclosure of Interest and Management of Conflicts of Interest Procedure	20-1	2020
5.	Managing and Investigating Potential Breaches of the JCU Code for the Responsible Conduct of Research Procedure	20-1	2020
6.	Peer Review Procedure	19-1	2019
7.	Publication and Dissemination of Research Procedure	20-1	2020
8.	Research Integrity Advisor Procedure	20-1	2020
9.	Research Supervision Procedure	20-1	2020
**U9**	**Macquarie University**		
1.	Authorship Standard	1.0	2020
2.	Collaborative Research Standards (interim)	Unavailable	Unavailable
3.	Conflict of Interest Standards (interim)	Unavailable	Unavailable
4.	Code for the Responsible Conduct of Research	2.0	2019

No.	Document	Version	Year
5.	Open Access Policy	Unavailable	2021
6.	Peer Review Standard	1.0	2020
7.	Research Code Complaints, Breaches & Investigation Procedure	1.0	2019
8.	Publication and Dissemination Standards (interim)	Unavailable	Unavailable
9.	Research Data Management Standards (interim)	Unavailable	Unavailable
10.	Supervision of Students Undertaking Research Standards (interim)	Unavailable	Unavailable
11.	Higher Degree Research Supervision	Unavailable	Unavailable
12.	Open Access Policy	Unavailable	2008
**U10**	**Monash University**		
1.	Activities Involving Genetically Modified Organisms Policy	1.0	2009
2.	Research Authorship and Attribution Policy	2.1	2020
3.	Conflict of Interest Procedure	9.1	2020
4.	Ethical Research and Approvals: Human Research Ethics Committee Procedures	1.0	2009
5.	Ethical Research and Approvals: Research Involving Human Participants Procedures	1.0	2009
6.	Ethical Research and Approvals Policy	1.1	2009
7.	Ethical Research and Approvals: Scientific Activities Involving Animals Procedures	1.1	2009
8.	Ethics Statement Policy	2	2018
9.	Integrity and Respect Policy	2.1	2020
10.	Intellectual Property: Commercialisation Revenue Sharing Provisions and Dispute Resolution Policy	2.0	2018
11.	Research Data Management Policy	1.2	2017
12.	Research Data Management: HDR Candidates Procedures	1.0	2020
13.	Research Data Management: Staff, Adjuncts and Visitors Procedures	1.1	2017
14.	Responsible Conduct of Research Policy	1.0	2011
**U11**	**Murdoch University**		
1.	Animal Ethics Policy	Unavailable	2020
2.	Conflict of Interest Policy	Unavailable	2020
3.	Human Research Ethics Policy	Unavailable	2020

No.	Document	Version	Year
4.	Intellectual Property Regulations	Unavailable	2017
5.	Public Interest Disclosure Policy	Unavailable	2019
6.	Recordkeeping Policy	Unavailable	2018
7.	Research Integrity Policy	Unavailable	2020
**U12**	**Queensland University of Technology**		
1.	Management of contracts, deeds and memoranda of understanding	Unavailable	2019
2.	QUT Staff Code of Conduct	Unavailable	2020
3.	Disciplinary action for misconduct and serious misconduct – senior staff	Unavailable	2009
4.	Conflict of Interest	Unavailable	2020
5.	Student Research Misconduct Committee charter	Unavailable	2018
6.	Research governance framework	Unavailable	2020
7.	Trade controls for goods, software, technology and services	Unavailable	2020
8.	QUT Code for responsible conduct of research	Unavailable	2020
9.	Managing and investigating potential breaches of the QUT Code for responsible conduct of research	Unavailable	2019
10.	Management of research data and primary materials	Unavailable	2020
11.	Intellectual Property	Unavailable	2020
12.	University Human Research Ethics Committee charter	Unavailable	2018
13.	University Biosafety Committee charter	Unavailable	2018
14.	University Animal Ethics Committee charter	Unavailable	2018
15.	QUT Student Code of Conduct	Unavailable	2020
16.	Open access for QUT research outputs (including theses)	Unavailable	2018
17.	Copyright	Unavailable	2017
18.	Information privacy	Unavailable	2020
**U13**	**RMIT University**		
1.	Animal Ethics Process	1.3	2020
2.	Authorship of Research Outputs Policy process	Unavailable	2016
3.	Conflict of Interest Declaration and Management Procedure	Unavailable	2020
4.	Conflict of Interest Policy	Unavailable	2020
5.	Disseminating Research Outputs Policy process	Unavailable	2017
6.	Human Research Ethics Procedure	Unavailable	2020

No.	Document	Version	Year
7.	Intellectual Property Policy	Unavailable	2020
8.	Management of Breaches of Research Integrity Procedure	Unavailable	2020
9.	Research Centres Policy Process	Unavailable	2017
10.	Research Data Management Policy process	Unavailable	2016
11.	Research Funding from the Tobacco Industry Policy process	Unavailable	2016
12.	Research Involving Genetically Modified Organisms (GMOs) Policy process	Unavailable	2016
13.	Research Policy	Unavailable	2020
**U14**	**The University of Adelaide**		
1.	Research Misconduct Procedure	Unavailable	2019
2.	Responsible Conduct of Research Policy	Unavailable	2019
3.	Research Student Handbook	Unavailable	2021
4.	Authorship Policy	Unavailable	2019
5.	Behaviour and Conduct Policy	Unavailable	2020
6.	Code of Conduct	Unavailable	Unavailable
7.	Conflict of Interest Procedure	2.0	2018
8.	University of Adelaide Enterprise Agreement 2017-2021	Unavailable	2017
9.	Fraud and Corruption Control Policy	Unavailable	2018
10.	Funding from the Tobacco Industry Policy	Unavailable	2021
11.	Intellectual Property Policy	Unavailable	2018
12.	Research Data and Primary Materials Policy	Unavailable	2016
13.	Student Misconduct Rules	Unavailable	2012
14.	Whistleblower Policy	Unavailable	2015
**U15**	**The University of Melbourne**		
1.	Authorship Policy	1	2013
2.	Management of Research Data and Records Policy	2	2013
3.	Research Integrity and Misconduct Policy	4	2020
4.	Graduate Research Training Policy	19	2020
5.	Supervisor Eligibility and Registration Policy	2	2020
6.	Research Ethics and Integrity	Unavailable	Unavailable
7.	Research Integrity principles	Unavailable	Unavailable
8.	Responsible research	Unavailable	Unavailable

No.	Document	Version	Year
**U16**	**The University of Queensland**		
1.	Information Management – Policy	Unavailable	2019
2.	Managing Complaints about the Conduct of Research – Procedures	Unavailable	2019
3.	Research Misconduct – Higher Degree by Research Students – Procedures	Unavailable	2019
4.	Responsible Conduct of Research – Policy	Unavailable	2019
5.	Schedule of sub-delegation – responsible conduct of research	Unavailable	Unavailable
6.	Student Integrity and Misconduct – Policy	Unavailable	2020
**U17**	**The University of Sydney**		
1.	Academic Honesty in Coursework Policy	Unavailable	2020
2.	Code of Conduct	Unavailable	2020
3.	EUnavailableternal Interests policy	Unavailable	2017
4.	Higher Degree by Research Supervision Policy	Unavailable	2020
5.	Intellectual Property Policy	Unavailable	2017
6.	Privacy Policy	Unavailable	2018
7.	Privacy Procedures	Unavailable	2019
8.	Public Comment Policy	2	Unavailable
9.	Recordkeeping policy	Unavailable	2018
10.	Reporting wrongdoing policy	Unavailable	2019
11.	Research agreements policy	Unavailable	2017
12.	Research Code of Conduct	Unavailable	2019
13.	Research Data Management Policy	Unavailable	2020
14.	Research Data Management Procedures	Unavailable	2020
15.	Student Charter	Unavailable	2020
16.	The University of Sydney Enterprise Agreement 2018-21	Unavailable	2018
17.	University of Sydney (Delegations of Authority) Rule 2020	Unavailable	2020
18.	University of Sydney (Student Appeals against Academic Decisions) Rule 2006	Unavailable	2020
19.	Working with children policy	Unavailable	2017
20.	Working with children procedures	Unavailable	2019

No.	Document	Version	Year
**U18**	**The University of Western Australia**		
1.	Authorship and Authorship Disputes procedures	Unavailable	2020
2.	Autonomous Sanctions Guideline	Unavailable	2020
3.	Defence Trade Controls Guidelines	Unavailable	2020
4.	Research Integrity Advisors Guideline	Unavailable	2020
5.	Research Integrity Guideline	Unavailable	2020
6.	Research Integrity Policy	Unavailable	2020
7.	University Policy on: Code of Conduct for the Responsible Practice of Research	Unavailable	2016
**U19**	**University of Canberra**		
1	Charter of Conduct and Values	Unavailable	2015
2.	Guidelines for the Management of Conflicts of Interest in Research	Unavailable	Unavailable
3.	Delegations of Authority Policy	Unavailable	2020
4.	Guidelines for Compliance with Regulation	Unavailable	Unavailable
5.	Guidelines for the Dissemination of Research Findings	Unavailable	Unavailable
6.	Higher Degree by Research Code of Practice	Unavailable	2013
7.	Higher Degree by Research Supervision Policy	Unavailable	2017
8.	Higher Degree by Research Supervision Policy	Unavailable	2020
9.	Management of Research Data and Primary Research Materiaks Policy	Unavailable	2017
10.	Peer Review of Research Policy	Unavailable	2014
11.	Procedures for Dealing with Complaints about Breaches of the Responsible Conduct of Research	Unavailable	2019
12.	Respect at Work (Prevention of Bullying) Policy	Unavailable	2016
13.	Responsible Conduct of Research Policy	Unavailable	2019
14.	Student Charter	Unavailable	Unavailable
**U20**	**University of New South Wales**		
1.	Animal Research Ethics Procedure	2.0	2018
2.	Code of Conduct	2.2	2017
3.	Complaint Management Policy	1.0	2018
4.	Conflict of Interest Policy	1.3	2017
5.	Fraud Policy	2.3	2018

No.	Document	Version	Year
6.	Higher Degree Research Supervision Policy	5.0	2020
7.	Higher Degree Research Supervision Procedure	2.0	2020
8.	Human Research Ethics Procedure	1.1	2017
9.	Intellectual Property Policy	2.3	2018
10.	Open Access Policy	1.0	2017
11.	Plagiarism Policy	2.0	2020
12.	Paid Outside Work by Academic Staff Policy	2.13	2017
13.	Delegations of Authority	3.5	2020
14.	Statement of authorship and location of data form	Unavailable	Unavailable
15.	Research Code of Conduct	2.0	2019
16.	Research Data Governance & Materials Handling Policy	1.0	2019
17.	Research Misconduct Procedure	2.0	2019
18.	Student Code of Conduct	3.0	2020
19.	Student Misconduct Procedure	3.0	2020
20.	University of New South Wales (Academic Staff) Enterprise Agreement	Unavailable	2018
21.	University of New South Wales (Professional Staff) Enterprise Agreement	Unavailable	2018
**U21**	**University of Newcastle**		
1.	Code of Practice for Higher Degree by Research Candidature	Unavailable	2018
2.	Collaborative Research and Ethics Approval Guideline	Unavailable	2019
3.	Conflict of Interest Policy	Unavailable	2020
4.	Intellectual Property Policy	Unavailable	2011
5.	Intellectual Property Procedure	Unavailable	2011
6.	Research Authorship Guideline	Unavailable	2015
7.	Research Breach Investigation Procedure	Unavailable	2018
8.	Research Data and Materials Management Guideline	Unavailable	2015
9.	Research Peer Review Guideline for Ethics Applications	Unavailable	2018
10.	Research Publication Responsibility Guideline	Unavailable	2015
11.	Responsible Conduct of Research Policy	Unavailable	2015
12.	Student Academic Integrity Policy	Unavailable	2011
13.	Student Conduct Rule	Unavailable	2015

No.	Document	Version	Year
**U22**	**University of Tasmania**		
1.	Research Policy	1	2020
2.	Research Training Policy	1	2020
3.	Intellectual Property Policy	1	2020
4.	Partnerships Policy	1	2020
5.	Compliance Policy	1	2020
6.	Academic Freedom and Free Speech Policy	1	2020
7.	Data and Information Governance Policy	1	2020
8.	Authorship of Research Procedure	4	2019
9.	Conflict of Interest Procedure	1	2017
10.	Management of Research Data Procedure	3	2019
11.	Managing Allegations of Research Misconduct Procedure	3	2017
12.	Responsible Conduct of Research Framework	Unavailable	2019
**U23**	**University of Technology Sydney**		
1.	Open Access Policy	1	2013
2.	Records Management Policy	1.1	2020
3.	Research Ethics and Integrity Policy	1.2	2018
4.	Research Management Policy	1.1	2018
**U24**	**University of Wollongong**		
1.	Academic Integrity Policy	9	2018
2.	Academic Misconduct (Coursework) Procedures	15	2020
3.	Authorship Policy	10	2017
4.	Code of Practice – Responsible Conduct of Research	11	2019
5.	Conflict of Interest Policy	11	2020
6.	Defence Trade Controls Guideline	1	2017
7.	Higher Degree Research (HDR) Student Academic Complaints Policy	6	2020
8	Higher Degree Research (HDR) Supervision and Resources Policy	19	2019
9.	Intellectual Property Policy	13	2020
10.	Open Access Policy	2	2017
11.	Research Data Management Policy	3	2019

No.	Document	Version	Year
12.	Research Misconduct and Complaints Management Procedure	1	2017
13.	Research Sponsorship and Collaboration Policy	5	2018
**U25**	**Western Sydney University**		
1.	Academic Staff Agreement 2017	Unavailable	2018
2.	Code of Conduct	Unavailable	2019
3.	Conflict of Interest Policy	Unavailable	2019
4.	Copyright Policy	Unavailable	2016
5.	Intellectual Property Policy	Unavailable	2012
6.	Open Access to Research Policy	Unavailable	2015
7.	Privacy Policy	Unavailable	2018
8.	Research Code of Practice	Unavailable	2016
9.	Research Data Management Policy	Unavailable	2015
10.	Student Code of Conduct	Unavailable	2020
11.	Student Misconduct Rule	Unavailable	2020
12.	Work Health and Safety Policy	Unavailable	2013
